# Development of a core evaluation framework of value-added medicines: report 2 on pharmaceutical policy perspectives

**DOI:** 10.1186/s12962-021-00296-2

**Published:** 2021-07-15

**Authors:** Zoltán Kaló, Zsuzsanna Ida Petykó, Frank-Ulrich Fricke, Nikos Maniadakis, Tomáš Tesař, Kateřina Podrazilová, Jaime Espin, András Inotai

**Affiliations:** 1 Center for Health Technology Assessment, Semmelweis University, Üllői rd. 25, 1085 Budapest, Hungary; 2 Pharmaceutical Policy Research, Syreon Research Institute, Mexikói str. 65/A, 1142 Budapest, Hungary; 3 Technische Hochschule Nürnberg, Nürnberg, Germany; 4 Department of Public Health Policies, Sector of Health Systems and Policy, School of Public Health, University of West Attica, Athens, Greece; 5 Department of Organisation and Management of Pharmacy, Faculty of Pharmacy, Comenius University in Bratislava, Bratislava, Slovakia; 6 Association of Health Insurance Companies, Prague, Czech Republic; 7 Andalusian School of Public Health, Granada, Spain

**Keywords:** Evidence, Generic reference pricing, Incremental innovation, Multi-criteria decision analysis, Drug repurposing, Value-added medicines, Value assessment framework

## Abstract

**Background:**

A core evaluation framework that captures the health care and societal benefits of value added medicines (VAMs, also often called repurposed medicines) was proposed in Report 1, aiming to reduce the heterogeneity in value assessment processes across countries and to create incentives for manufacturers to invest into incremental innovation. However, this can be impactful only if the framework can be adapted to heterogeneous health care financing systems in different jurisdictions, and the cost of evidence generation necessitated by the framework takes into account the anticipated benefits for the health care system and rewards for the developers.

**Areas covered:**

The framework could potentially improve the pricing and reimbursement decisions of VAMs by adapting it to different country specific decision-contexts such as deliberative processes, augmented cost-effectiveness frameworks or formal multi-criteria decision analysis (MCDA); alternatively, some of its domains may be added to current general evaluation frameworks of medicines. The proposed evaluation framework may provide a starting point for practices based on which VAMs can be exempted from generic pricing mechanisms or can be integrated into the reimbursement and procurement system, allowing for price differentiation according to their added value. Besides evidence from RCTs, pricing and reimbursement decision processes of VAMs should allow for ex-ante non-RCT evidence for certain domains. Alternatively, relying on ex-post evidence agreements—such as outcome guarantee or coverage with evidence development—can also reduce decision uncertainty.

**Conclusions:**

The core evaluation framework for VAMs could trigger changes in the existing pricing, reimbursement and procurement practices by improving the appraisal of the added value created by incremental innovation.

## Background

Pharmaceutical research and development (R&D) of new molecules entails substantial investment with usually over 10 years until patients can access the new products [1]. Current regulatory, pricing and reimbursement systems are structured to facilitate the market entry of originator products. More specifically, the development of orphan medicines has become the most attractive segment of pharmaceutical R&D in recent years, since—due to special public incentives in rare diseases with high unmet need—the clinical development period has been shortened and the failure rate of investigational target molecules has decreased [2, 3]. The case of orphan medicines indicates the significance of public interventions and incentives in socially important but neglected areas of pharmaceutical development.

Well-established medicines are pharmaceuticals with an active ingredient that has been used for several years and their efficacy and safety is already well known [4]. Incremental innovation (also known as continuous or evolutionary innovation) of medical therapies means the process of gradually improving these well-established medicines, devices and services. Value-added medicines (VAMs, also referred to as repurposed medicines) are created through the process of incremental innovation [5]. There are 3 major repurposing models used to create VAMs; (1) repositioning (refers to using the medicine in a new indication), (2) reformulation and (3) combination of established medicines or medicines with devices or with digital solutions. As shown by earlier contributions from both the authors and also by other researchers, (despite the relatively low development costs and reduced development timelines) repurposing established pharmaceuticals is a neglected area of pharmaceutical research and development, since the added value of incremental innovation may not be acknowledged by public policymakers [6, 7]. It should be highlighted that the price differential of VAMs compared to generic price levels is significantly less than the usual price premium of patented originator medicines; hence, their return-on-investment potential is more limited. If public decision-makers expected large scale pivotal randomized trials with long enough follow-up periods to justify all types of value claims for VAMs without additional regulatory incentives (e.g., longer data exclusivity), they would, in effect, block the incremental innovation on off-patent medicines [8]. A potential approach to facilitate additional research on established (i.e. off-patent) medicines would be to develop a core evaluation framework, which can provide guidance to manufacturers on what type of evidence generation should be considered in their development strategy. A proposal for such a core framework is presented in Report 1 [9]. The core framework consists of 11 individual value domains, including (1) Extending treatment options in a new indication with unmet medical need, (2) Individual needs/special needs of patient (sub)population, (3) Efficacy/Effectiveness, (4) Patient safety and tolerability, (5) Patient experience related to the therapy, (6) Adherence and Persistence, (7) Quality of life, (8) Patient’s economic burden, (9) Economic and health burden on informal caregiver, (10) Health care resource utilization, costs or efficiency and (11) Technological improvement with logistical considerations.

As the second part of this research, Report 2 discusses the key pharmaceutical policy implications of the proposed value assessment framework. The core evaluation framework is based on a systematic approach to facilitate the appraisal of differential value in a scientifically rigorous yet pragmatic way. Still, two important questions remain open. Firstly, more clarity is needed on whether the core evaluation framework can be transferred and adapted to heterogeneous health care financing systems. If reasonable solutions can be proposed for the framework adaptation in different jurisdictions—especially if best practices for the adaptation are shared—the willingness of decision-makers to use the value framework in their national pricing and reimbursement decisions may increase. Secondly, it should be explored whether the anticipated cost of evidence generation necessitated by the value framework takes into account the magnitude of claimed benefits for the health care system and the anticipated reward for the VAM developers. The objective of this review is to discuss different aspects related to these questions, mainly from the European perspective.

## Decision-making contexts for VAMs

Several countries have already introduced special evaluation criteria for special health technologies, such as orphan drugs, vaccines, end-of-life therapies, curative gene therapies or digital technologies. This trend indicates that a specific evaluation framework for a class of medical technologies can be politically attractive and is already happening in different jurisdictions.

If policymakers in several countries are interested in facilitating the sustainability of incremental pharmaceutical innovation, they can consider adapting the core evaluation framework of VAMs proposed in Report 1 to their national pricing, reimbursement or procurement system. As there are significant differences in the pharmaceutical policy processes across European countries, three main decision-making contexts are considered below for incorporating the core evaluation framework of VAMs to national policies.

### Deliberative decision-making process

Public payers in many countries apply policy tools to accelerate price competition of off-patent medicines by generic substitution or internal price referencing and select the more affordable generic or biosimilar medicines in the reimbursement or procurement system (i.e., through tendering). Certain medicines, including VAMs, could be exempted from generic substitution and price referencing groups, or they can be integrated into the reimbursement and procurement system with a reasonable price premium (compared to the reference generic medicines) due to their added value. In such cases, the exemption or the price premium could be decided in a deliberative process based on the submitted evidence or arguments. Due to the deliberative nature of the process, often no explicit rules and criteria are determined upfront; hence decisions may not be replicable, especially for external stakeholders. A core evaluation framework, that is detailed in Report 1, can provide guidance on the rules of exemption from internal price referencing or the acceptability of a price premium in the reimbursement or procurement process for all stakeholders concerned by the deliberation, including the decision-makers, which can eventually improve the consistency and replicability of decisions in deliberative decision frameworks.

### Augmented cost-effectiveness framework

The proposed core evaluation framework for VAMs can also be relevant in countries where cost-effectiveness analysis is mandated to justify the price premium of any medicine (e.g., VAMs compared to non-value-added generic pharmaceuticals). In such decision contexts, traditionally, the ratio of incremental costs and incremental quality-adjusted life years (QALY) is compared to a willingness-to-pay threshold. As the traditional cost per QALY assessment has a limited focus on patient experience, societal benefits and other non-traditional value propositions, an augmented cost-effectiveness analysis might be considered [10]. In an augmented analysis, non-traditional value elements can be aggregated either (1) in the benefits (e.g., improved patient experience) or (2) in the costs (e.g., reduced economic burden on patients and caregivers) or (3) they can modify the baseline willingness to pay threshold (e.g., equity considerations related to those VAMs, which can address the special needs of vulnerable patient subpopulations). From the technical perspective, each proposed value domain of VAMs can be considered among the benefits, costs or threshold modifiers; therefore, the core evaluation framework is applicable for augmented cost-effectiveness analyses.

### Multi-criteria decision analysis

There is a growing interest in applying specific extended evaluation frameworks and multi-criteria decision analysis (MCDA) to support the priority setting, pricing, reimbursement and procurement decisions of special types of health technologies, including orphan medicines, off-patent medicines, vaccines, medical devices or intensive care for patients with severe infections [11–19]. MCDA is a methodology for explicit appraisal of different alternatives by aggregating individual and often conflicting criteria into a single overall score, allowing to transform ad hoc decisions into transparent and replicable processes [20, 21]. In the future, some countries may incentivize investment into value-added innovation on established medicines by converting the core evaluation framework into a specific MCDA tool (analogously to those MCDA tools already used for specific health technologies) for repeated use in pricing, reimbursement or procurement decisions of VAMs. During the national adaptation of the core value framework, weights and scoring functions of each criterion should be elicited and the decision rule has to be established [22]. The development of a generalizable scoring function may not be an easy task for certain domains, e.g., in the patient experience criterion, as there is no consensus in the international health policy arena on how to define and measure improved patient experience [23].

### Extension of general evaluation frameworks

Still, in some countries, the separation of a new class of pharmaceutical products for policy decisions cannot be an attractive option from a political point of view. However, even in these countries, some domains of the core framework could be added to current “one size fits all” evaluation frameworks of medicines to make them more receptive to the benefits offered by VAMs. It is also expected that policymakers and health technology assessment (HTA) bodies will be more and more willing to consider non-RCT evidence or ex-post evidence generation techniques (described in the next chapter) for certain value claims of VAMs. Such an extension of general evaluation frameworks would be beneficial not only for VAMs but also for other special health technologies, such as personalized medicines, vaccines or medical devices.

## Complexity of evidence generation for the value domains

It is a fair expectation of policymakers, health care professionals and patients that the benefit of VAMs should be substantiated by robust scientific evidence. On the other hand, the evidence base of different health technologies is not equally strong for technology assessment before policy decisions. Although evaluators can hardly rely on evidence from double-blind, randomized controlled trials (RCTs) in the evaluation of medical devices, public health interventions or even orphan drugs, these technologies and interventions are still continuously incorporated into the health care systems with public reimbursement [24–27].

The situation is quite similar to the incremental innovation of established medicines. Firstly, VAM developers compose regulatory submissions by relying on the substantial existing knowledge about the safety and efficacy of the originator medicine and—depending on the claimed benefit— generate additional evidence that can support their application for reimbursement. Phase 2 and 3 RCTs are not necessarily required for the market authorization of several VAM types. Even when RCTs are required, due to the known safety profiles of established medicines in other patient populations, the follow-up period or the sample size can be reduced. Therefore, the strength of regulatory data for VAMs may not be comparable to the data of originator medicines. Consequently, the evidence expectations for reimbursement decisions on VAMs should be cognizant of the degree of innovation, the level of risk for the payers, and the feasibility of generating the expected level of evidence. Furthermore, evidence requirements for VAMs should be assessed on a case-by-case basis. Ideally, the cost of evidence generation should be proportionate to the magnitude of claimed benefits, anticipated risks and the expected price premium. The joint relative effectiveness assessment process (facilitated by EUnetHTA) provides an opportunity to judge the value also of VAMs in high priority diseases at the European level. These joint assessment processes may reduce the heterogeneity of quantifying expected benefits and reduce parallel efforts; generating evidence for a health technology concurrently in several countries [28–30].

Secondly, certain benefits, such as improved adherence, reduced resource utilization and treatment costs, can only be measured in the real-world setting and not in protocol-driven clinical trials. Compared to the cost of prospective RCTs, confirmatory real-world evidence for prescription drugs with a sufficient sample size could be collected at relatively low costs in observational studies, patient registries, or payers’ databases, typically ex-post, after the positive reimbursement decision [31]. This means that real-world evidence ex-ante (before policy decisions) can be generated only in early adopter countries, which may generate further questions about the transferability of real-world evidence. If we expect societal benefits from the timely and affordable incremental innovation of established medicines, a policy solution is needed that allows to fill the evidence gaps in certain value domains (e.g., improved patient adherence) without requesting time and resource-consuming RCTs from VAM developers.

Performance-based risk-sharing approaches with ex-post (after policy decisions) real-world data collection can provide a solution to the problem of premature scientific evidence in areas with a high unmet medical need [32]. If the VAM manufacturer provides a money-back guarantee or there is a planned revision of the original pricing and reimbursement decision based on the real-world value of the medicine, payers have reduced risks in accepting the interpretation of manufacturers about the magnitude of claimed benefits. Conditional coverage with evidence development may also be an appropriate policy approach to reduce the decision uncertainty. However, implementation of such agreements requires sufficient human resources and are also subject to other conditions, including (1) the availability of an objective measure for the expected benefit, which (2) cannot be manipulated by any stakeholders, and (3) are available within the routinely collected data (e.g., claims databases) or can be collected with minor incremental costs, and finally, (4) it can be reviewed or audited by both partners of the agreement. In addition, the collected ex-post evidence should be considered not only a public good but also as a global public good for the benefit of patients or even HTA doers in other countries [33]. Due to these conditions, coverage with evidence development schemes may not be applicable for the majority of VAMs.

The complexity of evidence generation and acceptability of evidence (i.e., RCT, non-RCT, real-world evidence) for decision-makers are different for each domain in the core evaluation framework of VAMs. Table 1 provides an overview of perceived costs and complexity of evidence generation for each domain, with a special focus on which domains can be addressed more easily with ex-ante and ex-post data collection. These initial judgments, however, should be validated in the future by surveying patients, industry representatives and policymakers.

**Table 1:** Initial judgement on the acceptability of evidence and complexity of evidence generation for each value domain in the core evaluation framework

Method of data collection	Source	Value domains										
		Unmet medical need		Health gain (measured by health care professionals)		Patient reported outcomes			Burden on households		Burden on health care systems	
		Extending treatment options in new indication with unmet medical need	Individual needs/special needs of patient (sub)population	Efficacy/ effectiveness	Patient safety and tolerability	Patient experience related to the therapy	Adherence and Persistence	Quality of life	Patient’s economic burden	Economic and health burden on informal caregiver	Health care resource utilization, costs or efficiency	Technological improvement with logistical considerations
Ex-ante	Evidence from RCT	+ + + / €€€	+ + / €€€	+ + + / €€€	+ + / €€€	+ / €€€	NA	+ + + / €€€	NA	+ + + / €€€	NA	NA
	Non-RCT evidence (e.g., observational study, patient registry, single-arm trial)	+ + / €	+ + + / €	+ / €	+ + + / €	+ / €€	+ + / €€	+ / €€	+ / €€	+ + / €€	+ / €€	+ / €
Ex-post	Real-world evidence (e.g., coverage with evidence development, outcome guarantee)	NA	+ + / €	+ + / €	+ + / €	+ + / €€	+ + + / €	+ + + / €€	+ + + / €€	+ + + / €€	+ + + / €	+ + / €

(+ / +  + / +  +  + : Perceived acceptability from the decision maker's perspective, €/€€/€€€: Perceived complexity and cost of data collection from the manufacturer’s perspective, RCT—randomized controlled trial)

## Incentives for incremental innovation

Currently, there are limited incentives for pharmaceutical companies to invest in further improving health technologies after patent expiry, despite the known targets for potential improvement (i.e., unmet medical need), minimal chances for unexpected serious adverse events and consequently lower development costs. As a consequence, the evidence base of non-patented medicines—which represent the vast majority of the therapeutic armamentarium—hardly improves over time. Although international policymakers and regulators can make the motion for necessary changes (e.g. the forthcoming HORIZON-HLTH-DISEASE-2021-04-02 call to build a European innovation platform for the repurposing of medicinal products), this unfavorable trend can hardly be solved without policy actions and better coordination at a national level in most countries [34].

The first option may be to develop predictable and transparent mechanisms to implement value-based price differential for VAMs relative to their generic comparators. Alternatively, VAMs may be exempted from certain policy solutions to facilitate generic price competition in those countries where this is not a common practice yet. We hope that the publication of the core evaluation framework proposed in Report 1 could be an important trigger for changes in the existing pricing, reimbursement and procurement practices by proposing an explicit list of potential domains for determining the added value of incremental innovation.

A typical example for the inappropriate policy environment is that repositioned medicines in their new indications or extended patient populations may be subject to internal price referencing or generic substitution at the pharmacy level. Even if this is not officially allowed, but in the real-world the exemption rule is not enforced to prevent the off-label use of generic medicines in new indications or patient populations, there is limited incentive for the private investment in drug repurposing. Therefore, the exemption rule should be supported by further regulatory and technical solutions or specific pathways to prevent the free-ridership of other manufacturers, who keep the development cost of their generic medicines at the minimum level.

As an alternative to private investment by VAM manufacturers, public investment or public-private partnership could also be utilized to test off-patent medicines in new indications or patient populations, as exemplified by recent trials of hydroxychloroquine to prevent or treat the SARS-CoV‐2 (or COVID-19) infection [35–37]. However, these cases are fairly rare for two reasons. Firstly, significant public investment (i.e., cost of pivotal trials) to extend the evidence base of existing medicines is allocated only during public health crises with significant economic externalities, mainly pandemics. Secondly, if the public investment is made at a national level, free-ridership also exists by other countries that can benefit from the innovation without making the investment. Still, large international public programs, such as Horizon Europe or Innovative Health Initiative or non-governmental organizations, such as the European Organisation for Research and Treatment of Cancer (EORTC), the Anticancer Fund, the International Myeloma Foundation (IMF) and the Bill and Melinda Gates Foundation may be able to allocate funds for additional research of established medicines for the sake of societal benefits without the need for a direct financial return on investment [38]. The United States as the most significant pharmaceutical market, alone would be sufficiently large to accommodate a national public program to support the repurposing of generic medicines, as recently proposed by Conti et al. [39]. However, even if sufficient public investment is made in different jurisdictions, manufacturers are necessary partners during the process of product launch.

Increased investment into the development of VAMs may positively impact different stakeholders, primarily patients, but also health care systems and manufacturers. However, changing the landscape of pharmaceutical R&D processes, specifically VAM regulations and policies, should be continuously monitored to prevent any negative outcomes. Special attention should be given to situations where market competition is limited and market concentration exists prior to or upon the introduction of VAMs to the market. The objective of such surveillance mechanisms could prevent issues, such as unjustified price increases or potential shortages of medicines.

## Future perspectives

The core evaluation framework for VAMs (see Report 1) can only deploy its full impact if it is adapted and adopted to the national pricing, reimbursement or tendering systems in several countries in the upcoming years. Similarly to the EUnetHTA HTA Core Model, the national derivate of the core VAM evaluation framework can differ from country to country [28–30]. Out of the three potential decision contexts, the standardization of rules for exemption from internal price referencing or acknowledgment of a fair price premium for a reasonable differential value is the easiest approach to how the proposed value framework can be utilized in the majority of countries. As the utilization of MCDA in health policy decisions has constantly been increasing [11–19], the core value framework may be converted into an MCDA tool with some additional work, especially in countries with political and economic interest to strengthen the R&D activities of local generic and biosimilar manufacturers [20]. Discussions about augmented cost-effectiveness frameworks have only started recently at international conferences. Therefore, it may take some time to reach consensus and to develop robust methods for integrating the core evaluation framework into such a concept.

The core evaluation framework gives a message to VAM developers that it is not enough to complete the minimal technical and regulatory steps of repositioning, reformulating or combining established medicines. They should also consider how to facilitate the policy-relevant value judgment on their medicines by ex-ante (i.e., RCT or non-RCT) or ex-post (i.e., real-world) evidence generation. In other words, the complexity of data collection should not be an excuse to approach payers or HTA bodies without a sound evidence base for the differential value [40].

On the other hand, authors feel that further research is needed to clarify the complexity, feasibility and costs of evidence generation in particular value domains. The initial overview presented in Table 1 is just one of the first steps in a multistakeholder dialogue foreseen in the coming years. Lessons should be learned from the pilot cases in those countries, where decision-makers would be interested in being pioneers in the adaptation of the core value framework to utilize the benefits of incremental innovation.

## Conclusions

Implementation of the core evaluation framework has multiple policy implications. The national adaptation of the framework across countries can reduce the heterogeneity of pricing, reimbursement or procurement decision-making practices related to VAMs on a broader international level. The framework may guide manufacturers on evidence requirements of VAMs, which eventually can facilitate the global evidence generation strategy for VAMs from their early development phase through to post marketing surveillance. Overall, improved predictability, transparency, consistency and reduced heterogeneity of decision-making could contribute to a more attractive and predictable business model for investing in VAMs and finally, resulting in the availability of more patient-centered and affordable medicines on the market.

## Data Availability

All data generated or analysed during this study are included in this published article [and its Additional files].

## Abbreviations

EORTC: European Organisation for Research and Treatment of Cancer
HTA: Health technology assessment
IMF: International Myeloma Foundation
MCDA: Multi-criteria decision analysis
QALY: Quality-adjusted life years
R&D: Research and development
RCT: Randomized controlled trials
VAM: Value-added medicines
